# A Safety Limit of the Number of Artificial Canals That Can Be Prepared by Two Rotary Endodontic Files Operated at Two Different Speeds: A Novel Approach

**DOI:** 10.3390/bioengineering12090985

**Published:** 2025-09-17

**Authors:** Omar Alzahrani, Khalid Merdad, Tariq Abuhaimed, Zuhair S. Natto, Amna Y. Siddiqui, Osama S. Alothmani

**Affiliations:** 1 Department of Advanced General Dentistry, Faculty of Dentistry, King Abdulaziz University Dental Hospital, Jeddah 21589, Saudi Arabia; oaaalzahrani@kau.edu.sa; 2 Department of Endodontics, Faculty of Dentistry, King Abdulaziz University, Jeddah 21589, Saudi Arabia; kmerdad@kau.edu.sa; 3 Department of Restorative Dentistry, Faculty of Dentistry, King Abdulaziz University, Jeddah 21589, Saudi Arabia; tabuhaimed@kau.edu.sa; 4 Department of Dental Public Health, Faculty of Dentistry, King Abdulaziz University, Jeddah 21589, Saudi Arabia; znatto@kau.edu.sa

**Keywords:** fatigue life of NiTi files, file separation, RPM

## Abstract

Utilizing a novel approach that concomitantly assessed cyclic fatigue and torsional overloading, we aimed to establish the maximum number of artificial canals that can be prepared by Hyflex EDM and NeoNiTi A1 operated at two different speeds until their separation. Forty-eight files were equally divided into four groups: (A) Hyflex EDM operated at 300 rotations per minute (RPM) and (B) at 500 RPM, and (C) NeoNiTi A1 at 300 RPM and (D) at 500 RPM. Files were used to completely shape 10 sequential artificial canals unless file separation occurred. Maximum number of canals prepared was noted and averaged. Fractography was conducted to determine the mechanism of file separation. Hyflex EDM prepared significantly more canals compared to NeoNiTi A1 (*p* = 0.008). When operated at 300 RPM, Hyflex EDM prepared significantly more canals compared to NeoNiTi A1 (*p* = 0.028), whereas no significant difference was observed when they were operated at 500 RPM (*p* = 0.116). One NeoNiTi file broke due to cyclic fatigue while another one separated due to torsional overloading. Hyflex EDM files showed signs of both mechanisms. Within the limitations of this study, one file safely prepared four to five canals before its fracture. There was a trend towards fewer prepared canals as the RPM increased.

## 1. Introduction

In 1988, Walia et al. introduced nickel titanium (NiTi) endodontic files by grinding orthodontic wires [1]. In comparison to stainless steel (SS) files, NiTi files were more flexible, exhibiting a low separation frequency, resulting in a more predictable shaping of curved root canals [2]. Engine-driven NiTi files better preserved the original canal anatomy when compared to manual SS files, resulting in fewer procedural concerns, less canal transportation, and prepared root canals more quickly decreasing intra-operative time [3,4]. Significantly higher chances for favorable outcomes after non-surgical root canal treatment using modern instrumentation approaches, including engine-driven NiTi instruments, were reported in comparison to instrumentation with SS hand files [5]. Nevertheless, engine-driven NiTi files are liable to separate consequent to cyclic fatigue or torsional overloading [6]. Many in vitro studies have been carried out to assess the cyclic fatigue and torsional overloading of engine-driven NiTi files. These models employed for separation assessment have included clamping devices, artificial metal tubes, or extracted teeth with different angles of curvature trying to simulate clinical conditions [7,8,9]. It has been difficult to transfer all the clinical variables that affect the performance of engine-driven NiTi files to in vitro settings. The most ideal model would be testing the behavior of engine-driven NiTi files in natural teeth. Instrumented canals, however, cannot be re-prepared because the canal course changes during instrumentation [9]. Obtaining natural teeth with identical morphology is another problem [10]. There are no international standards for engine-driven NiTi files testing in the literature [9].

Extrapolation of the results of laboratory findings to clinical practice is challenging because the in vitro methodologies and designs vary greatly from actual clinical instrumentation scenarios [8,11]. The clinical value of the number of cycles to fracture (NCF), torque at fracture, or angle of rotation at fracture is limited. The literature is replete with studies evaluating the two mechanisms of NiTi instrument fracture and the factors influencing them. However, most of these studies independently evaluated these two mechanisms, even though files may clinically fail because of both mechanisms occurring concomitantly [9,12]. The clinical validity of cyclic fatigue testing is questionable [7]. The same applies to assessment of torsional resistance [13]. Therefore, development of new methods to assess the performance of NiTi files is needed. If such methods define a safety limit, i.e., the number of canals that can be safely prepared by one file before being discarded, such findings might serve as a more applicable guide for daily clinical practice.

Electrical Discharge Machining (EDM) is one of the proposed methods for improving the mechanical properties of NiTi files. It is a contactless thermal erosion method producing electrically conductive materials. The surface of the NiTi alloy is melted, leaving an eroded surface behind. The instrument is heated from 10 min to 5 h at 300 to 600 °C, with ultrasonic cleaning before and after the procedure, as well as with an acid [14]. Currently, there are two rotary file systems in the market manufactured using EDM technology: the Hyflex EDM (Coltene/Whaledent, Cuyahoga Falls, OH, USA) and the NeoNiTi A1 (NEOLIX, Chatres-la-Foret, France). Both systems received considerable attention and have been extensively tested.

Hyflex EDM exhibited a prominent resistance to cyclic fatigue compared to the original Hyflex controlled memory (CM) files [15]. EDM processing boosted the mechanical performance of Hyflex EDM compared to Hyflex CM [16]. Hyflex EDM demonstrated significantly higher NCF than ProTaper Gold, ProTaper Universal [17], Reciproc, WaveOne [18], OneShape, WaveOne Gold, Reciproc Blue [19], V-Taper 2, V-Taper 2H, Hyflex CM, and ProTaper Next X2 [20]. It also revealed a significantly longer fatigue life compared to TRUShape [21]. Hyflex EDM’s resistance to torsion was superior to V-Taper 2H, Hyflex CM, and ProTaper Next X2 [20], but inferior to ProTaper Gold and ProTaper Universal [17]. Another study reported that the torque at fracture endured by Hyflex EDM was significantly lower than Recirpoc and WaveOne, while its angle of rotation at fracture was significantly higher [18].

NeoNiTi A1 is another rotary system made of a special alloy that has been treated with an EDM mechanism to produce sharper edges and better flexibility. It possesses non-homothetic rectangular sections, along with the blades, and should be used at 300–500 RPM with 1.5 Ncm torque setting [22]. NeoNiTi A1 exhibited significantly higher NCF than Mtwo, Twisted file, ProTaper Next X2 [23], Reciproc [24], F360, F6 SkyTaper, iRace, One Shape, ProTaper Next, Reciproc, Revo-S, WaveOne Gold [25], EdgeFile X3, and Neoendo Flex [26]. A recent multi-analytical study reported that the resistance of NeoNiTi to cyclic fatigue was higher than ProTaper Next and ProTaper Universal, similar to OneCurve, but lower than ProTaper Gold and EdgeTaper Platinum. Considering resistance to torsion, ProTaper Gold, EdgeTaper Platinum, and ProTaper Universal demonstrated higher endurance compared to NeoNiTi, while OneCurve and ProTaper Next performed similar to it [27].

Three studies compared several parameters of NeoNiTi and Hyflex EDM [25,28,29]. Both systems exhibited a similar resistance to cyclic fatigue [25,28,29]. Hyflex EDM exhibited a higher resistance to torsion in one study [29], but performed similar to NeoNiTi in the other [28]. The metallurgic properties of both systems were comparable [28,29]. They acted alike in terms of shaping the canals of maxillary molars [29] and mandibular molars [28].

Despite the extensive assessment of HyfleX EDM and NeoNiTi in the reported literature, all these studies assessed resistance to cyclic fatigue or torsional overloading in separate set-ups. NiTi files are subjected to simultaneous cyclic and torsional stresses when they are used for shaping root canals [9,12]. Furthermore, none of the previous studies evaluated the effect of operating Hyflex EDM and NeoNiTi at a lower speed (300 RPM) or higher speed (500 RPM) on their performance. The operational RPM impacted the mechanical performance of rotary NiTi files [12].

The aim of this study was to evaluate if there was any difference in the number of simulated canals that could be prepared with Hyflex EDM (25/~) and NeoNiTi A1 (25/0.06) files before their separation when operated at two different RPMs, utilizing an approach that concomitantly takes the impact of cyclic fatigue and torsional overloading into account. The null hypothesis was that there was no significant difference in the number of simulated canals prepared by either Hyflex EDM or NeoNiTi until their fracture when operated at 300 and 500 RPM.

## 2. Materials and Methods

### 2.1. Sample Specifications

The present study used a total of 480 clear polyester resin transparent simulated root canals (Endo Training Block 02 taper, REFA 0177; Dentsply Maillefer, Ballaigues, Switzerland). All canals had a taper of 0.02, a 40° angle of curvature, a 5 mm radius of curvature, and apical foramen diameter of 0.15 mm. Canals were 17 mm long, with a 12 mm straight section and a 5 mm curved section. The canals were assigned randomly to four equal groups with 120 canals per group. The simulated root canals in each group were shaped using either NeoNiTi A1 25/06 or Hyflex EDM (25/~) rotary files as follows:

Group A: Hyflex EDM files (*n* = 12) at 300 RPM and torque of 2.5 Ncm.

Group B: Hyflex EDM files (*n* = 12) at 500 RPM and torque of 2.5 Ncm.

Group C: NeoNiTi A1 files (*n* = 12) at 300 RPM and torque of 1.5 Ncm.

Group D: NeoNiTi A1 files (*n* = 12) at 500 RPM and torque of 1.5 Ncm.

Each rotary file was used to completely shape 10 simulated canals unless separation occurred.

### 2.2. Preparation of the Simulated Canals

A SS K-file size 10 (Kerr, Romulus, MI, USA) was inserted in each simulated canal until its tip became visible at the apical foramen. The stopper was adjusted to reference point, the file was withdrawn and measured, and the working length was set at 0.5 mm shorter than that length. Rotary files were attached to the 16:1 contra-angle head of the Tri Auto ZX2 cordless motor handpiece (J Morita, Kyoto, Japan) with torque limits and RPM set according to the group investigated. All blocks were instrumented in a standardized manner by one experienced operator operating the file in three up and down pecking strokes of 2 mm amplitude until the full working length was reached. Patency was maintained throughout the procedure by inserting a size 10 K-file 1 mm longer than the working length. The instrumentation of the simulated canal was considered complete when a matching cone reached the full working length with a tug-back. The operator shifted to the next simulated canal and the shaping procedure was repeated as mentioned earlier. This was performed until separation occurred, or until all 10 canals were completely shaped without any separation. The number of canals completely shaped was recorded for each group and the mean ± standard deviation was calculated.

### 2.3. Irrigation Protocol

Irrigation with 5.25% sodium hypochlorite (NaOCl) was performed with a 27-gauge side-vented needle (Endo-Irrigation Needle; Transcodent, Neumunster, Germany) attached to a 10 mL syringe using positive pressure technique with the needle inserted 2 mm short of the working length. This was performed after using each rotary and patency file.

### 2.4. Fractography

One separated file from each group was photographed using a stereo microscope under 50× magnification (RaySmart Technology Co., Ltd., Shenzhen, China), and then the fractured surface was scanned using a scanning electron microscope (Aura100, Seron Technologies, Gyeonggi-do, Republic of Korea) to identify the type of separation.

### 2.5. Statistical Analysis

SPSS version 22 software (IBM Corp, Somers, NY, USA) was used to conduct the statistical analysis. The impact of the file type (Hyflex EDM vs. NeoNiTi A1) and RPM (300 vs. 500 RPM) on the number of shaped simulated canals before separation was compared using an independent sample *t*-test. A one-way ANOVA was used to assess the impact of the file type (Hyflex EDM vs. NeoNiTi A1) and RPM (300 vs. 500 RPM) at the same time, in relation to number of prepared canals before separation. A two-way ANOVA was also used to measure impact of the file type (Hyflex EDM file vs. NeoNiTi A1 file) or speed (300 vs. 500 RPM) separately in relation to the number of prepared canals before separation. Level of significance was set at *p* < 0.05.

## 3. Results

In total, Hyflex EDM prepared significantly more canals (5.25 ± 1.65) before separation in comparison to NeoNiTi A1 (3.96 ± 1.57) (*p* = 0.008). When divided according to the operational RPM, Hyflex EDM significantly prepared more canals (5.83 ± 1.85) than NeoNiTi A1 (4.25 ± 1.42) before fracture when files were operated at 300 RPM (*p* = 0.028). There was no significant difference between the two systems operated at 500 RPM (*p* = 0.116) in terms of the number of canals prepared before fracture, with Hyflex EDM preparing an average 4.67 ± 1.23 canals compared to 3.67 ± 1.72 canals by NeoNiTi A1. There was no statistically significant difference in the number of canals prepared before fracture between Hyflex EDM operated at 500 RPM and 300 RPM (*p* = 0.083) or NeoNiTi A1 operated at 500 RPM and 300 RPM (*p* = 0.376).

Regardless of the file type, the mean number of prepared canals until separation at 300 RPM was 5.04 ± 1.81. The mean number of prepared canals until separation at 500 RPM was 4.17 ± 1.55. This difference was not significant (*p* = 0.078).

Based on Table 1, one-way ANOVA revealed a significant difference between the groups (*p* < 0.001), and a post hoc test with Bonferroni correction identified that Hyflex EDM operated at 300 RPM prepared significantly more canals when compared to NeoNiTi A1 at 500 RPM (*p* = 0.012). When the file type (Hyflex EDM vs. NeoNiTi A1) and speed (300 vs. 500 RPM) were compared in relation to the number of simulated canals prepared before separation, a two-way ANOVA revealed no significant difference (*p* = 0.434); see Table 1.

**Figure 1:**
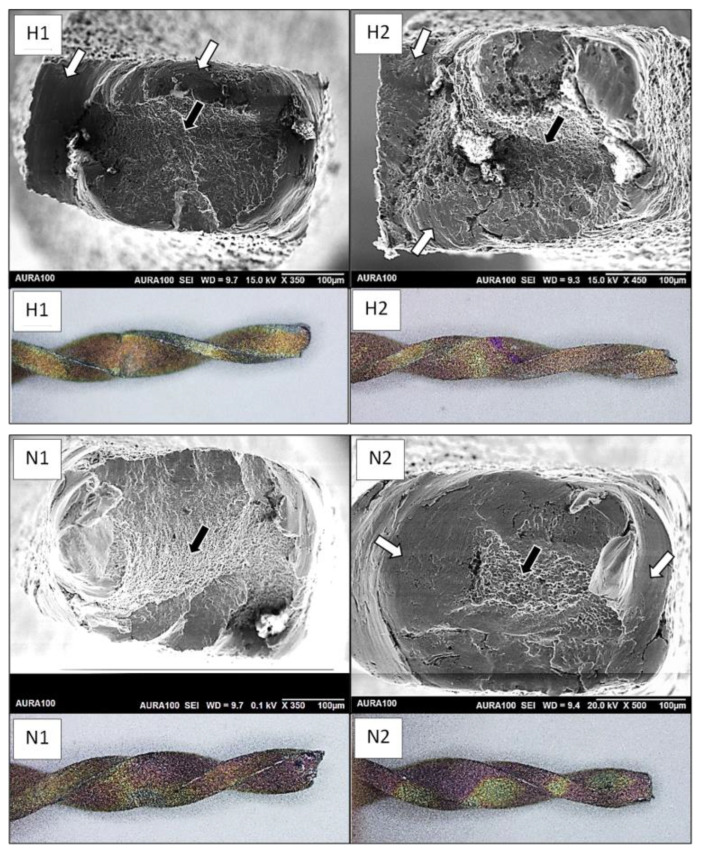
Images of SEM and stereomicroscope of fractures files. Profiles images of all files showed no plastic deformation. SEM images of (H1) and (H2) showed circular abrasion marks (white arrows) indicating torsional overloading, along with striations and dimples at the periphery (black arrows) indicating cyclic failure. SEM images of (N1) showed predominant signs of cyclic fatigue, while (N2) exhibited typical central dimples surrounded by circular abrasions, indicating torsional overloading. H1: Hyflex EDM Group A, H2: Hyflex EDM Group B, N1: NeoNiTi A1 Group C, N2: NeoNiTi Group D.

Under the stereo microscope examination (Figure 1), all files fractured without plastic deformation, indicating a brittle fracture that typically represented separation due to cyclic fatigue. However, when the files were examined under SEM, the NeoNiTi file from Group C (Figure 1, N1) only showed signs of cyclic fatigue, while the other file representing Group D (Figure 1, N2) only showed signs of torsional overloading. However, both Hyflex EDM files (Figure 1, H1 and H2) revealed signs of erosion marks, as well as dimples and striations, indicating a combined torsional overloading and cyclic failure.

## 4. Discussion

Given the current need for developing new assessment methods for the mechanical performance of engine-driven NiTi files that can be easily translated to clinical application, we aimed to determine the maximum number of canals that a single file could safely prepare (i.e., without file separation) before being discarded. The main advantage of our approach was in its ability to assess the durability of NiTi files when subjected to simultaneous torsional and cyclic stresses while shaping root canals. Under standardized irrigation conditions, simulated resin canals were completely shaped using Hyflex EDM (25/~) or NeoNiTi A1 (25/06) files operated at two different RPMs. In comparison to NeoNiTi, Hyflex EDM prepared significantly more canals, indicating that Hyflex EDM was more fracture-resistant with higher durability. The safety limit (i.e., number of canals that can be prepared by one file before discarding it) was unaffected by the RPM. Within the limitations of this study, one file could safely prepare four to five canals before being discarded. This could serve as a more clinically applicable guide for clinicians compared to the traditional NCF, torque at failure, and angle of rotation.

To ensure repeatability, standardized artificial canals made of resin with pre-defined parameters were used instead of extracted teeth. Obtaining a sufficient number of extracted teeth with similar morphological features is challenging [10]. Opting to include contralateral premolars to provide balanced groups reduced the available sample size to around 20% [30]. However, these results might have been affected by their employment of CBCT parameters for the final matching assessment. When the more robust microtomographic assessment was used, a considerable number of comparable contralateral premolars [31] and mandibular incisors [32,33] were collected. Being a novel methodology, we chose to perform the current study using the readily available plastic models. However, we intend to conduct a future study that includes anatomically matched extracted teeth based on microcomputed tomography parameters. Such an approach will improve the generalizability of the obtained results.

It should be noted that the standardized anatomy of resin blocks may have influenced our findings because canals in vivo have different diameters and trajectories, resulting in different stresses on the files. Another confounding factor to consider is the difference in hardness between resin and dentin [34]. Shaping plastic blocks might lead to plastic melting from the generated frictional heat leading to a higher liability of instrument separation [35]. Future studies should consider using 3D printed teeth possessing mechanical properties comparable to radicular dentin, while allowing for testing the performance of the NiTi files in variable anatomical complexities [34]. This should expand the applicability of the obtained results.

The resistance of WaveOne Gold and Reciproc to cyclic fatigue was significantly reduced when testing was conducted under immersion in 5% NaOCl compared to testing in ambient air [36]. The results of another study that tested the cyclic fatigue of K3, K3XF, and Vortex files did not find such effect [37]. A recent meta-analysis reported that resistance to cyclic fatigue might be reduced when testing is performed under immersion in heated NaOCl, and that this effect was more pronounced on files made of conventional NiTi wires [38]. To the best of our knowledge, none of the previous studies investigated the effect of NaOCl on the performance of NeoNiTi. However, only one study evaluated the effect of NaOCl on the mechanical performance of Hyflex EDM and found that NCF was significantly reduced under immersion in 3% NaOCl compared to when testing was performed in ambient air [39]. It must be pointed out that the full immersion of teeth and shaping files in NaOCl does not occur clinically. It is unlikely that our results were affected by irrigation with 5% NaOCl maintaining a wet environment for instrumentation. The effect of NaOCl on the mechanical performance of Hyflex EDM and NeoNiTi A1 deserves further investigations.

Studies assessing the cyclic fatigue of engine-driven NiTi under static conditions and not considering the effect of environmental temperature were considered as having a very low clinical value. Dynamic testing mimics clinical usage to a greater extent [7]. Our proposed methodology includes a pecking motion that simulates clinical situations. This dynamic testing increased the validity of the reported results. Our choice to conduct our experiment under ambient air was because testing the resistance of Hyflex EDM to cyclic fatigue at different temperatures showed that the fatigue life of the files was not affected by increasing the environmental temperature [21,40]. Nonetheless, the effect of environmental temperature on the performance of NeoNiTi A1 is unknown. Considering the similar differential scanning calorimetry graphs of Hyflex EDM and NeoNiTi [28,29], it can be assumed that the effect of environmental temperature on the performance of NeoNiTi will be similar to Hyflex EDM. In fact, the austinite-finish temperature of new NeoNiTi files and those used and sterilized ranged between 52.36 ± 1.07 °C and 53.40 ± 1.09 °C, which was much higher than body temperature. This signified the presence of considerable martensite crystalline structures at room and body temperatures, indicating the high flexibility of the NeoNiTi files [35]. The effect of environmental temperature on the performance of Hyflex EDM and NeoNiTi A1 should be further evaluated.

The results of the current study showed that Hyflex EDM safely prepared significantly more canals compared to NeoNiTi A1 (*p* = 0.008), indicating the higher durability of Hyflex EDM. Previous reports showed that both systems exhibited similar metallurgic composition and comparably shaped maxillary and mandibular molars with equivalent resistance to cyclic fatigue [25,28,29]. It is difficult to relate the current results with previous reports due to the integral disparities in the methodologies and reported parameters. The observed higher durability of Hyflex EDM in the current study could be attributed to its cross-section, variable taper, and its operation on a higher torque, as all these factors have been reported to affect the performance of engine-driven NiTi files [8,11,41,42].

The manufacturer of Hyflex EDM claims that the files regain their original shape upon autoclaving [43]. Only two studies have evaluated the effect of autoclaving on the fatigue life of Hyflex EDM, and reported conflicting results [44,45]. The former study found that repetitive autoclave cycles reduced the resistance of Hyflex EDM to cyclic fatigue, while the latter found no such effect either on resistance to cyclic fatigue or resistance to torsional overloading. None of the previous studies have evaluated the effect of autoclaving on the performance of NeoNiTi A1 files. Nevertheless, the regaining of the original shape upon autoclaving is not a feature of NeoNiTi A1. Hence, the results of the current study must be viewed with consideration to our decision to shape 10 consecutive canals without intermediate sterilization.

The clinical use of rotary files more than four times increased the separation incidence from 0.3% to 7% [46]. However, the included studies differed in the definition of number of uses, with some identifying it based on the number of teeth, while others reported the usage based on the number of canals. The results of the current study showed that four to five canals can be safely prepared before disposal of the files. This might be considered as a short fatigue life. However, it must be pointed out that the files in the current study were used without being preceded by any coronal flaring or proper glide path establishment. Such steps might reduce the stresses on the shaping files leading to longer durability and further shaping of more canals. Furthermore, the safe preparation of four to five canals resembles the single use of the files. This was associated with a 0.33% clinical separation rate [46].

Different studies have evaluated the effect of RPM on the incidence of file separation and reported conflicting results. Operating files at higher RPMs had a greater chance for separation compared to operating them at lower RPMs [12,47]. Another study reported that files rotated at 350 RPM deformed half as much as those driven at 150 RPM, implying that the higher RPM was safer and allowed for a prolonged use of the files [48]. Alteration of RPM had no effect on resistance to cyclic fatigue [49]. To the best of our knowledge, this is the first study to evaluate the effect of different RPMs on the performance of Hyflex EDM and NeoNiTi. Results showed that the variation in RPM did not affect the number of prepared artificial canals, although there was a trend towards a reduction in the number of prepared canals with increased RPM (*p* = 0.083 and 0.376 for Hyflex EDM and NeoNiTi A1, respectively). It is possible that the pecking motion providing dynamic movement implemented in the current study had a damping effect over the influence of RPM, unlike the usual static continuous rotation of the files adopted in the cyclic fatigue models used in previous studies.

Earlier studies classified the type of failure by the presence or absence of plastic deformation, where the absence of plastic deformation indicated cyclic failure [6]. Later studies used SEM images of fractured cross-sections to identify the type of failure [50]. Circular marks are signs of torsional failure, while striations and dimples are signs of cyclic failure. However, it has been reported that torsional failure can occur without plastic deformation under repeated torsional loading [51]. The results of the current study reported no plastic deformation on the profile images of the files. However, SEM images showed that some files had signs of torsional failure (Figure 1). It seems that, in some files, both stresses contributed to the fracture of the file. Variation in the mechanism of separation depicted in our results strengthened the proposed approach to assess the resistance of engine-driven NiTi files to fracture.

## 5. Conclusions

When compared to NeoNiTi A1 files, Hyflex EDM had a higher durability, enabling the safe shaping (i.e., without separation) of significantly more canals. RPM had no effect on fracture resistance of both systems, though there was a trend toward fewer prepared canals as RPM was increased. Within the limitations of this study, one file safely prepared four to five canals before its fracture. More studies are needed to establish a more realistic “clinical safety limit” for engine-driven NiTi files using this novel approach.

## Figures and Tables

**Table 1:** Comparison of the impact of file type and RPM on the number of prepared simulated root canals before separation.

Group	Total Files	*p* Value *	*p* Value **	No. of Canals Prepared Until Separation	*p* Value *	*p* Value **
Hyflex EDM RPM 300	12	<0.001 *	0.434	5.83 ± 1.85 ^a^	0.012 *	0.525 **
Hyflex EDM RPM 500	12			4.67 ± 1.23		
NeoNiTi A1 RPM 300	12			4.25 ± 1.42		
NeoNiTi A1 RPM 500	12			3.67 ± 1.72 ^a^		

* One-way ANOVA *p* < 0.05 considered statistically significant. ** Two-way ANOVA *p* > 0.05 considered statistically not significant. ^a^ Post hoc *p* < 0.05 considered statistically significant.

## Data Availability

The original contributions presented in this study are included in the article. Further inquiries can be directed to the corresponding authors.
